# Length and orientation constancy learning in 2-dimensions with auditory sensory substitution: the importance of self-initiated movement

**DOI:** 10.3389/fpsyg.2015.00842

**Published:** 2015-06-17

**Authors:** Noelle R. B. Stiles, Yuqian Zheng, Shinsuke Shimojo

**Affiliations:** ^1^Shimojo Psychophysics Laboratory, Biology and Biological Engineering, California Institute of TechnologyPasadena, CA, USA; ^2^Shimojo Psychophysics Laboratory, Computation and Neural Systems, California Institute of TechnologyPasadena, CA, USA; ^3^Mechanical Engineering, California Institute of TechnologyPasadena, CA, USA

**Keywords:** sensory substitution, audition, vision, blind, multisensory

## Abstract

A subset of sensory substitution (SS) devices translate images into sounds in real time using a portable computer, camera, and headphones. Perceptual constancy is the key to understanding both functional and phenomenological aspects of perception with SS. In particular, constancies enable object externalization, which is critical to the performance of daily tasks such as obstacle avoidance and locating dropped objects. In order to improve daily task performance by the blind, and determine if constancies can be learned with SS, we trained blind (*N* = 4) and sighted (*N* = 10) individuals on length and orientation constancy tasks for 8 days at about 1 h per day with an auditory SS device. We found that blind and sighted performance at the constancy tasks significantly improved, and attained constancy performance that was above chance. Furthermore, dynamic interactions with stimuli were critical to constancy learning with the SS device. In particular, improved task learning significantly correlated with the number of spontaneous left-right head-tilting movements while learning length constancy. The improvement from previous head-tilting trials even transferred to a no-head-tilt condition. Therefore, not only can SS learning be improved by encouraging head movement while learning, but head movement may also play an important role in learning constancies in the sighted. In addition, the learning of constancies by the blind and sighted with SS provides evidence that SS may be able to restore vision-like functionality to the blind in daily tasks.

## Introduction

One class of sensory substitution (SS) devices encodes an image into tactile or auditory stimuli to allow vision-like perception after the onset of blindness. The vOICe device is an auditory SS device in which vertical position is translated into pitch, horizontal position is translated into scan time (encoded in stereo), and brightness is translated to loudness. Several studies have shown that both blind and sighted users of SS devices, such as the vOICe, can obtain the functional aspects of vision via crossmodal plasticity (Bach-Y-Rita et al., 1998; Renier et al., 2005; Amedi et al., 2007; Poirier et al., 2007a; Proulx et al., 2008; Plaza et al., 2009; Striem-Amit et al., 2012).

Learning via sensory-motor interactions and dynamic sensory processing has been indicated as valuable in SS studies as well (Epstein et al., 1989; Segond et al., 2005; Poirier et al., 2006; Proulx et al., 2008; Siegle and Warren, 2010; Brown et al., 2011; Levy-Tzedek et al., 2012; Haigh et al., 2013). In particular, Siegle et al. showed that distal attribution, or perception of object external space, can be perceived with a tactile SS device (Siegle and Warren, 2010). Furthermore, Proulx et al. showed that daily-life sensory-motor experience with auditory SS in addition to experience during tests, improved SS capabilities relative to no training beyond tests (Proulx et al., 2008). Ward and Wright discussed the sensorimotor similarities and differences between SS perception and traditional visual perception and the concept of “embodiment,” or incorporation of the SS camera into bodily perception (Ward and Wright, 2014).

Further, in general, active, as opposed to passive, interactions with the environment have been proven to be more effective for sensory-motor learning (Held and Hein, 1963). Some argue even more strongly that active interaction is crucial to visual awareness (O'Regan and Noe, 2001). Gibson's classical concepts such as “dynamic, direct perception” or “picking up higher-order invariance from the input affordance” may also be relevant (Gibson, 1950). Reafferent signals, or feedback from motor commands, have been hypothesized by Mountcastle to provide a memory-based prediction to optimize sensory-motor learning (Mountcastle, 1978). Such reafferent signals may critically enhance SS spatial perception and therefore constancy.

Nevertheless, perceptual constancy, which is the ability to perceive a feature as constant despite dynamic visual changes, has not yet been quantified with SS. Constancies are a valuable part of perception, and are important to functional task performance as well as accurate environmental perception (*e.g.*, externalization). There are several types of visual constancies such as size constancy (constant size independent of distance), shape constancy (constant shape independent of object rotation), orientation constancy (constant object orientation independent of head tilt), position constancy (constant object position independent of head and body movement), and brightness constancy (constant brightness independent of external illumination) (Rock, 1983). This study investigates if two types of visual constancies (length and orientation constancy) can be learned using the vOICe SS device with sighted and blind users. In particular, we will investigate the 2D versions of length and orientation constancy, which are a subset of the 3D versions. In vOICe the 2D constancies are important to perception yet challenging to learn (due to reasons detailed below) and therefore we argue can be considered as valuable elements of the family of constancy types.

Orientation constancy may be considered a basis for object externalization by allowing the object angle to be perceived as the orientation angle of an independent, *external* object in real-world coordinates, thereby enabling adaptive behavior. In other words, visual orientation constancy allows the angle of an object to be determined independent of head tilt, and is useful for determining an object's angle relative to the direction of gravitational force (Day and Wade, 1969) (Note: Head tilt can occur in 3 dimensions, however we will test only the left-right planar tilt in this paper). Visual orientation constancy is generated by the visual frame of reference as well as by proprioceptive information that includes head orientation, and kinesthetic feedback, allowing for the correct interpretation of tilted images due to head tilting (Neal, 1926; Witkin and Asch, 1948; Day and Wade, 1969). Orientation constancy would be particularly useful to blind SS users, as it would allow them to determine object stability via the object's tilt, independent of their own locomotion/movement. Important mobility information conveyed by orientation constancy would include the stability of tables, chairs, and other furniture, as well as natural objects such as rocks, trees, and branches. Orientation constancy is also useful for obstacle avoidance of objects (especially when leaning), whose position in space (determined via orientation constancy) is critical to locomotion around them.

Length constancy (the ability to estimate length independent of object angle), studied here in 2-dimensions rather than in 3-dimensions, is a sub-type of shape constancy (the ability to estimate object shape independent of viewing angle) (Epstein and Park, 1963; King et al., 1976; Norman et al., 1996). In the normal visual system, line length can be estimated by means of the same type of neural computation at any 2D angle. Length constancy in 2D is particularly difficult to learn with the vOICe SS device, due to the vOICe image-to-sound encoding scheme (Meijer, 1992), which makes lines of different orientation angle (angle here refers to the orientation in the 2D plane of the line) but the same length perceptually and computationally different (*i.e.*, a vertical line is a short sound burst of a wide pitch range where length is determined by the pitch range, a horizontal line is a longer sound of a smaller pitch range where length is determine by sound duration). Thus the vOICe style of V-to-A translation created a new domain of constancy challenge and will indicate the flexibility of perceptual processing if it can be learned. In addition, it has been shown that the vertical-horizontal illusion, where vertical lines are perceived to be longer than horizontal lines of the same length, occurs while using SS in sighted but not blind users (Renier et al., 2006). This illusion will make length constancy even more challenging. Shape constancy is also critical for both the recognition of objects that are viewed from different perspectives as they are rotated in 3D space, and for the estimation of spatial size as a 2D object is rotated. Length constancy in 2D space enables the categorization of objects via size, and the correct estimation of spatial location horizontally and vertically. Further, length and shape constancy allow an object to be recognized not as changing identity after rotation, but rather as a cohesive single object in the environment. This allows for the object to be externalized in space as a real singular object. Therefore, not only is 2D length constancy counterintuitive to learn with the vOICe device, but it is also critical to performing daily localization and recognition tasks with the device.

If constancies can be learned, the potential for SS users to attain a high level of vision-like functional capability (as outlined above) as well as for SS to behave as a perceptual modality (like vision or audition alone) would be considerably enhanced. We hypothesize that the vOICe auditory SS device can be used by both the sighted and blind to learn orientation and length constancy tasks. We also believe that this learning could be amplified by dynamic interaction with stimuli, and thereby provide an insight into how visual-motor experiences shape perceptual constancies in general.

## Materials and methods

### Participants

Twelve blindfolded sighted and four blind participants (three late blind, one congenital) were trained on the vOICe device for at least 8 days at approximately 1 h per day performing three evaluation tasks (orientation constancy task, length constancy task, and a localization task). All reported experiments were approved by the Caltech Internal Review Board, and all subjects provided informed consent. Sighted participants were recruited via the Caltech Brainscience subject recruitment website (http://brainscience.caltech.edu/). Blind subjects were recruited through previous participant contacts. Blind participant training was identical to the sighted training except for extending the number of training sessions to 10 sessions from 8 sessions (1 h/day) and using tactile control tasks instead of visual control tasks.

### Training structure

Training with the vOICe device was performed on consecutive days at a black felt covered table. Each session included the following tasks (in this order): length constancy, orientation constancy, and localization. Data was recorded for each task. The localization task data is not included herein, as it was employed primarily as a training element. These initial training tasks were followed by additional training for the remaining time in the hour. The additional training started with simple object centering and shape identification in the first session, followed by extended length or orientation constancy training in the following sessions. Length constancy training involved estimating the lengths of lines at just one orientation angle at a time (such as just 90° lines) and the orientation constancy training involved estimating angles with the head at only one tilt.

### Orientation constancy task

**Figure 1:**
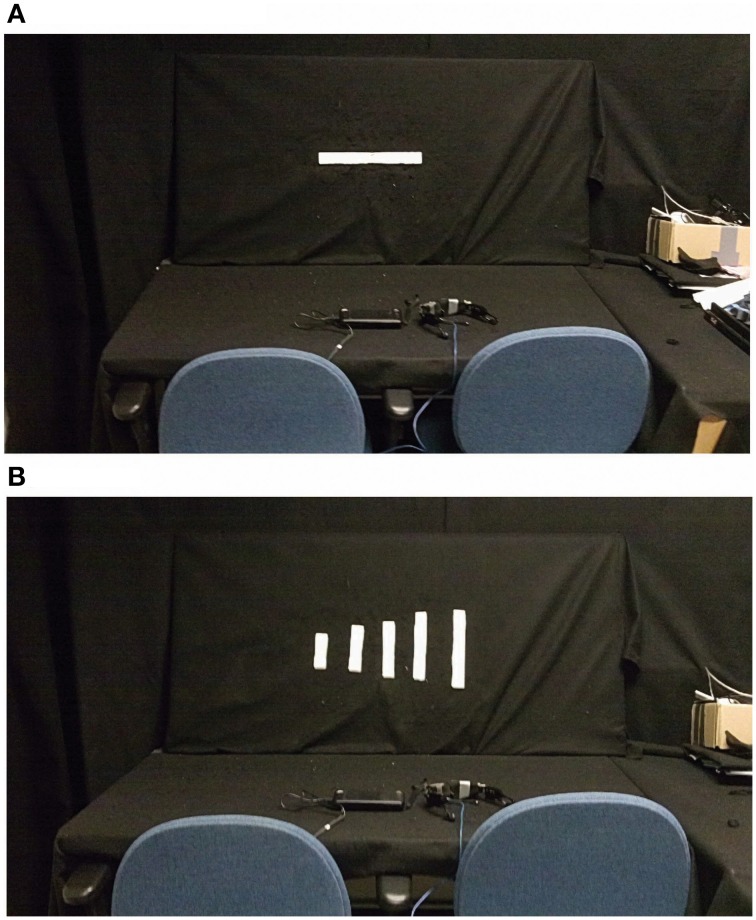
**Experimental Setup. (A)** Shows the experimental setup for the orientation constancy task (classification of line orientation angle independent of head tilt; the horizontal line shown is 90°). To perform the orientation constancy task, the subject sits in the right chair and determines the angle of the line placed on the board in one of six orientations (vertical, horizontal, −22, 22, −45, and 45° relative to the vertical; 6AFC). **(B)** Shows the experiment setup for the length constancy task (classification of line length independent of line orientation angle). To perform the length constancy task, the subject sits in the right chair and determines the length of the line placed on the board in one of four orientations (vertical, horizontal, −45, and 45° relative to the vertical; 5AFC) (during each task only one bar is placed on the board).

To evaluate orientation constancy, participants were presented with a bar (3 × 30 cm) at 6 different angles (6AFC: 0, 90, 45, −45, 22, or −22° relative to vertical; clockwise rotations correspond to positive angles) with three potential head positions (vertical, tilted left, or tilted right) while using the vOICe device, and then were asked to determine the orientation angle of the bar (Figure 1A). The experimenter placed the bar on a black felt covered wall in front of the seated participant and visually estimated each angle position to be presented to the participant. Participants were told to tilt their head left, right, or vertical (no tilt), and were permitted to determine the head tilt angle that they were most comfortable using in each trial (provided that their head was stationary). One head position was requested for each trial. The subject was seated about 81 cm from the bar to be evaluated. The bar angles and head tilt positions (left, right, or vertical) were randomized for each session with 15 total trials per task performance. Participants performed the task once per session. No visual or tactile controls were performed. Before starting the task, participants were shown the angles at different head tilts; participants were also given feedback for each trial. If necessary, initially participants were given hints of the arithmetic process to account for head tilt in angle perception. As training progressed, the trainer attempted to reduce the number hints (if any were given) to the necessary arithmetic steps. For a small subset of participants, arithmetic was difficult and therefore a tactile bar was used to help learn the addition and subtraction of angles based on head tilt.

### Length constancy task

To evaluate length constancy, participants were presented with 5 lengths of bars (5AFC: 3 cm by either 9, 12, 15, 18, or 21 cm), while the bar was placed in one of four orientations (0, 90, 45, or −45° relative to vertical; clockwise rotations correspond to positive angles) (Figure 1B). Participants were asked to determine the length of the bar presented independent of the angle that it was presented at. The subject was seated about 81 cm from the bar to be evaluated. Participants first performed the task with the vOICe device (original task) and then with vision (touch for the blind; control task) in each session. The bar lengths and angles were randomized in order for each session, which included 20 trials for each task performance (original and then control). Head tilt instructions varied across subjects (further information is provided in the length constancy training details section). Before starting the training, participants were briefly shown the line lengths in a subset of the angles. Participants were given feedback for each trial, and if necessary shown an example line length following a trial as a reminder.

### The vOICe device

Participants used a vOICe device to learn the constancy tasks. The vOICe device uses a camera embedded in a pair of sunglasses or a webcam attached externally to glasses. Sighted participants were requested to close their eyes during training and evaluation, and wore opaque glasses and/or a mask to block direct visual input. The camera provided a live video feed of the environment, and a small portable computer was used to encode the video into sound in real time.

The vOICe software was obtained online at seeingwithsound.com and was used for the video-to-sound encoding. Two blind participants were forced to transition from one camera and device setup to another setup part way through training due to device failure; their data did not indicate any difficulty with this transition. In principle, any technical differences in the vOICe device setup would not make any difference in terms of training efficiency and task performance, except for possible minor differences in spatial perception due to the gain of the camera, camera field of view, and camera placement. In particular, field of view changes between cameras may alter the absolute size of the line however we did not want participants to memorize the sound durations (or another auditory feature) for each of the bars, and re-iterate that memorized element. Rather, we wanted participant to learn how to interpret vOICe and learn how to identify different line lengths at different angles (as is done in vision).

The training sessions were video recorded for later data analysis. The participants were informed of the recording and consented to it.

### Length constancy training details

**Figure 2:**
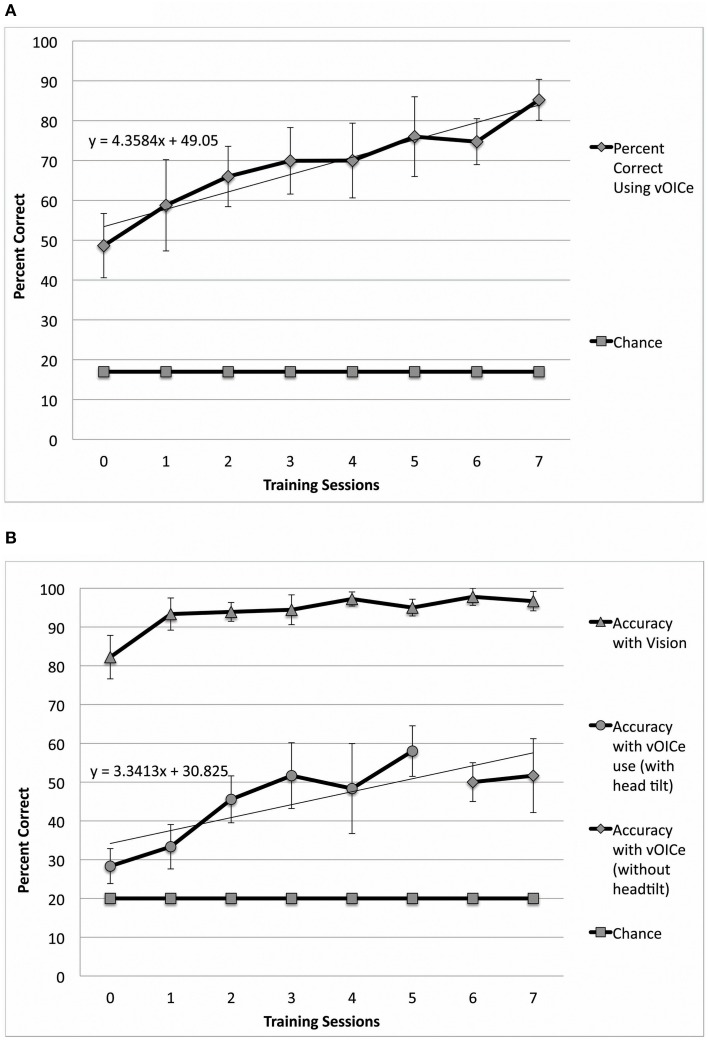
**Performance in both constancy tasks by sighted subjects** (***N*** **= 9 – 10)**. In **(A)** the orientation constancy task performance (classification of line orientation angle independent of head tilt) is plotted as a function of the number of training sessions (*N* = 10). In **(B)** the length constancy task performance (classification of line length independent of line orientation angle) is plotted as a function of the number of training sessions (*N* = 9). Error bars represent the one standard deviation for both plots. (Note: See methods for subject inclusion and exclusion for each plot).

**Figure 3:**
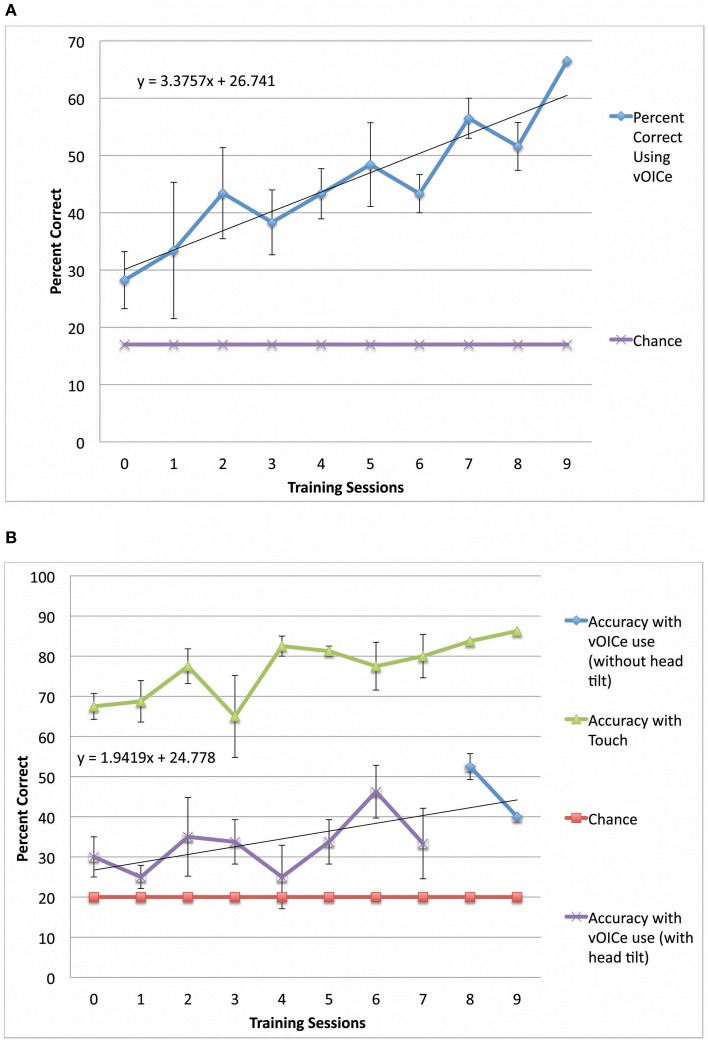
**Performance in both constancy tasks by blind subjects** (***N*** **= 4). (A)** Plots the task performance of orientation constancy as a function of the number of training sessions in the blind participants only (*N* = 4). **(B)** Plots the task performance of length constancy as a function of the number of training sessions in the blind participants only (*N* = 4). The error bars represent the one standard deviation for both plots.

**Figure 4:**
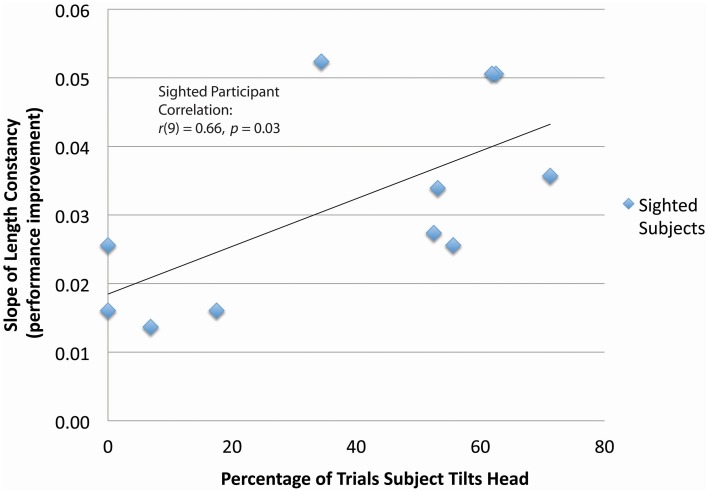
**Correlation between head tilt and performance improvement for the length constancy task** (***N*** **= 11)**. Significant correlation between head tilt and performance improvement is shown for the length constancy task (*N* = 11) [*r*_(9)_ = 0.66, *p* = 0.03]. (Note: See methods for subject inclusion and exclusion). The slopes of length constancy are the slopes of the plots of length constancy percent correct as a function of the training time for each subject. A larger positive slope corresponds to a larger performance improvement per training session.

Since our primary aim was to explore training style/design, participant training was varied to determine the optimal training procedures (Table 1). Two sighted participants were directed not to use head tilt during the length constancy task and were not included in Figure 2A or Figure 2B, but were included in **Figure 4** (Table 1, participants 10 and 11). One sighted participant was directed to use head tilt intermittently [4 out of 12 sessions with head tilt, Table 1 (participant 12)] and was excluded from Figure 2B, but was included in Figure 2A. The remaining participants were asked to tilt their head in the initial length constancy trials, but at the end of the training participants evaluated the bar length without head tilt (Table 1, participants 1–9). Figure 2B includes and excludes different participants in several data points so that the figure can show data for head tilt trials in Sessions 0–5, and no-head-tilt data in trials in Sessions 6–7 (Sessions 0–4 and 7 included 9 participants, Session 5 included 5 participants, and Session 6 included 7 participants). Figure 2B is based on data from sighted participants instructed to use head tilt in initial sessions and no head tilt in later sessions to show the retention of the learning gained during the head tilt sessions, which is important to our hypothesis that head-tilting aids learning. The same procedure was used for Figure 3B, which shows data from blind participants for head tilt in Sessions 0–7, and only data for no-head-tilt in Sessions 8–9 (Session 7 included 3 participants, and all other sessions included 4 participants). Due to technical constraints in the usage of the device, the desire for training exploration, as well as limitations due to less visual processing experience in recent years in blind participants, we were not able to carry out the experiments with only one simple and identical procedure across all participants.

Table 1**Head-tilting Sessions for vOICe Training on Length Constancy**.

**Table:** 

	**Session 1**	**Session 2**	**Session 3**	**Session 4**	**Session 5**	**Session 6**	**Session 7**	**Session 8**
**SIGHTED SUBJECTS**								
Participant 1	HT	HT	HT	HT	HT	HT	HT	NHT
Participant 2	HT	HT	HT	HT	HT	HT	HT	NHT
Participant 3	HT	HT	HT	HT	HT	HT	NHT	NHT
Participant 4	HT	HT	HT	HT	HT	HT	NHT	NHT
Participant 5	HT	HT	HT	HT	HT	HT	NHT	NHT
Participant 6	HT	HT	HT	HT	HT	NHT	NHT	NHT
Participant 7	HT	HT	HT	HT	HT	NHT	NHT	NHT
Participant 8	HT	HT	HT	HT	HT	NHT	NHT	NHT
Participant 9	HT	HT	HT	HT	HT	NHT	NHT	NHT
Participant 10	NHT	NHT	NHT	NHT	NHT	NHT	NHT	NHT
Participant 11	NHT	NHT	NHT	NHT	NHT	NHT	NHT	NHT
Participant 12	NHT	NHT	NHT	NHT	NHT	HT	HT	NHT

**Table:** 

	**Session 1**	**Session 2**	**Session 3**	**Session 4**	**Session 5**	**Session 6**	**Session 7**	**Session 8**	**Session 9**	**Session 10**
**BLIND SUBJECTS**										
Participant 13	HT	HT	HT	HT	HT	HT	HT	HT	NHT	NHT
Participant 14	HT	HT	HT	HT	HT	HT	HT	HT	NHT	NHT
Participant 15	HT	HT	HT	HT	HT	HT	HT	NHT	NHT	NHT
Participant 16	HT	HT	HT	HT	HT	HT	HT	NHT	NHT	NHT
**KEY**										
HT, Head Tilt Allowed in that Training Session										
NHT, No Head Tilt Allowed in that Training Session										

*This table lists all participants of the vOICe training and indicates in which sessions they were allowed to tilt their head*.

### Head tilt analysis

**Figure 5:**
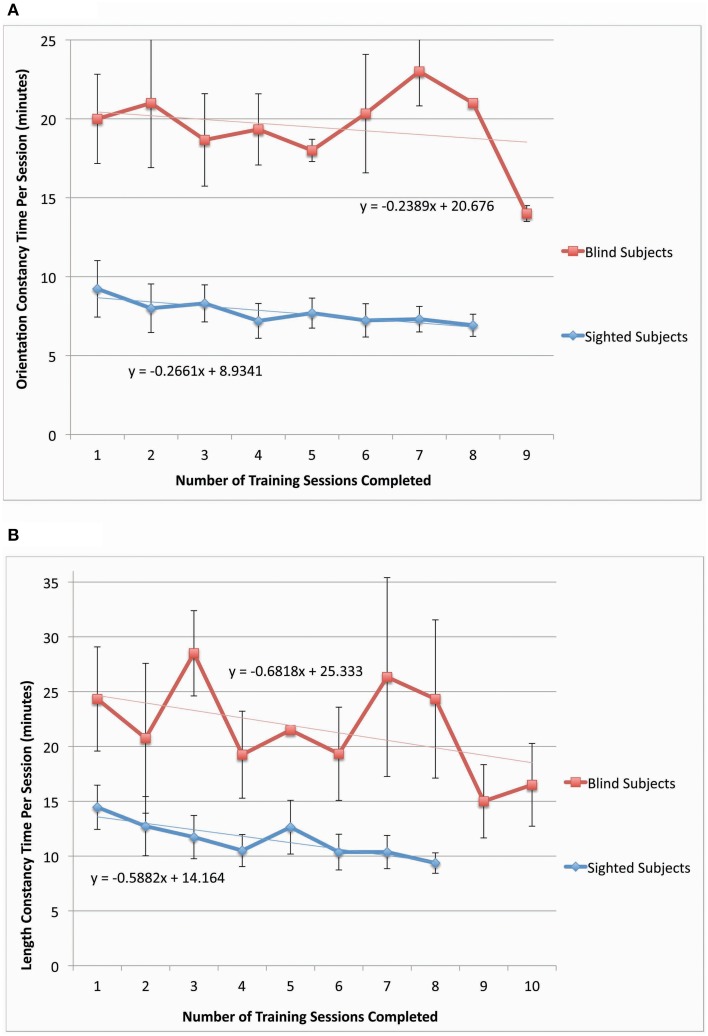
**Orientation and length constancy task duration for both sighted** (***N*** **= 11–12) and blind participants** (***N*** **= 3–4)**. The time per session for each participant was determined from video recordings of the training sessions and then averaged across the participants. The constancy task durations [orientation constancy for **(A)** and length constancy for **(B)**] are plotted as a function of the number of training sessions completed. In **(A)**, one blind participant was omitted from the data due to lack of video from three consecutive sessions. In **(B)**, one sighted participant was omitted due to an exploratory protocol that included four different transitions between head-tilt allowed trials and no-head-tilt allowed trials. Error bars represent the one standard deviation.

Data analysis of head tilt was performed using the video recordings of training sessions. Head tilt was quantified by counting the number of trials in which the participant used head tilt while exploring the stimulus, divided by the total number of trials (160 trials for sighted and 200 trials for blind), and multiplied by 100 to obtain a percentage. Head tilt was estimated for training sessions with missing video recordings (3.8% of all sessions had missing video recordings) by using the average head tilt for sessions of the same type for the same participant (such as head tilt allowed or head tilt not allowed sessions). Two participants were trained without any head tilt permitted in the training, and are included in the head tilt correlation (Figure 4) and time to decision (Figure 5) plots, but not the main data set on performance (Figures 2A,B) to exclude training inhomogeneity.

### Time to decision analysis

Data analysis on time to decision was performed on the video recordings of training sessions by recording the onset and end of a task during the training session for all training sessions of all training participants. Training sessions lacking a video recording were omitted from the analysis. One blind participant in the orientation constancy task was omitted from the time to decision data due to the lack of three consecutive training session videos.

### Kruskal–Wallis, correlation, and regression analyses

Kruskal–Wallis, correlation, and regression analyses were performed in MATLAB. Regression analysis was used to determine if the rate of a participant's improvement was significantly different from zero. The regression analyses used the regstats function. Kruskal–Wallis analysis was used to determine if two groups of data had significantly different slopes or intercepts (Kruskal–Wallis function). Correlation calculations were performed with the corr function.

## Results

Sighted and blind vOICe users were able to classify objective line angles independent of head tilt (orientation constancy), and to further improve with training (Figure 2A for the sighted, and Figure 3A for the blind). The rate of improvement was significant in both groups [Sighted (*N* = 10, 8 training sessions): *t*_(6)_ = 7.89, *p* = 0.0002; Blind (*N* = 4, 9 training sessions): *t*_(7)_ = 5.36, *p* = 0.001; regression analysis]. In addition, blind participants had an average slope that was not significantly different from that of the sighted, while the intercepts were significantly different [Slope: χ^2^(1, *N* = 4−10) = 1.62, *p* = 0.20; Intercept: χ^2^(1, *N* = 4−10) = 4.50, *p* = 0.03, Kruskal–Wallis).

Sighted and blind vOICe users were able to classify line length independent of angle (length constancy), and to further improve with training (Figures 2B, 3B). The rate of improvement was significant in both groups [Sighted (*N* = 9, 8 training sessions): *t*_(6)_ = 3.95, *p* = 0.008; Blind (*N* = 4, 10 training sessions): *t*_(8)_ = 2.72, *p* = 0.03; regression analysis]. Blind participants had an average slope of improvement and intercepts that were not significantly different from that of the sighted [Slope: χ^2^(1, *N* = 4−9) = 3.46, *p* = 0.06; Intercept: χ^2^(1, *N* = 4−9) = 1.17, *p* = 0.28, Kruskal–Wallis]. During head tilt allowed sessions, head tilting was encouraged by instruction. Improved line length classification correlated significantly with head tilt frequency during the task (Figure 4). To determine head tilt frequency, the number of trials in which participants tilted their head were counted for all task sessions from video recordings (the average number of head tilt trials was used for sessions with missing video). The percentage of trials in which head tilt was used was plotted against the slope of the participant's length constancy improvement (from the interpolated slopes in individual participant plots similar to Figure 2B). The sighted participants correlated length constancy task improvement with head tilt with *r*_(9)_ = 0.66, *p* = 0.03, whereas sighted and blind participant data combined had *r*_(13)_ = 0.60, *p* = 0.02. Adding the blind data to the sighted data may have reduced the correlation because of differences in training, and perception (Pasqualotto and Proulx, 2012). Participants' initial performance at the length constancy task (i.e., intercept) was not significantly correlated with head tilt frequency [for sighted and blind combined *r*_(13)_ = 0.08, *p* = 0.78]. The lack of correlation between initial performance and head tilt serves as a sanity check for a “prior bias” (i.e., those who had a better initial performance tended to tilt their head more).

The performance times in both constancy tasks decreased as training sessions progressed for both sighted and blind participants (the plot of time per session as a function of training session number had a negative slope for length and orientation constancy, blind data, and sighted data, as shown in Figures 5A,B). We also should note that this time per session data has the following potential sources of noise: time for answering questions or additional explanation, time to give participants additional examples of stimuli, and time for participant to locate the stimulus of interest in their field of view. The decrease in time to perform the training task indicates a tendency toward task automaticity and away from extensive top-down attention, thereby beginning to mimic the intuitive and automatic nature of perceptual constancies in the sighted.

**Figure 6:**
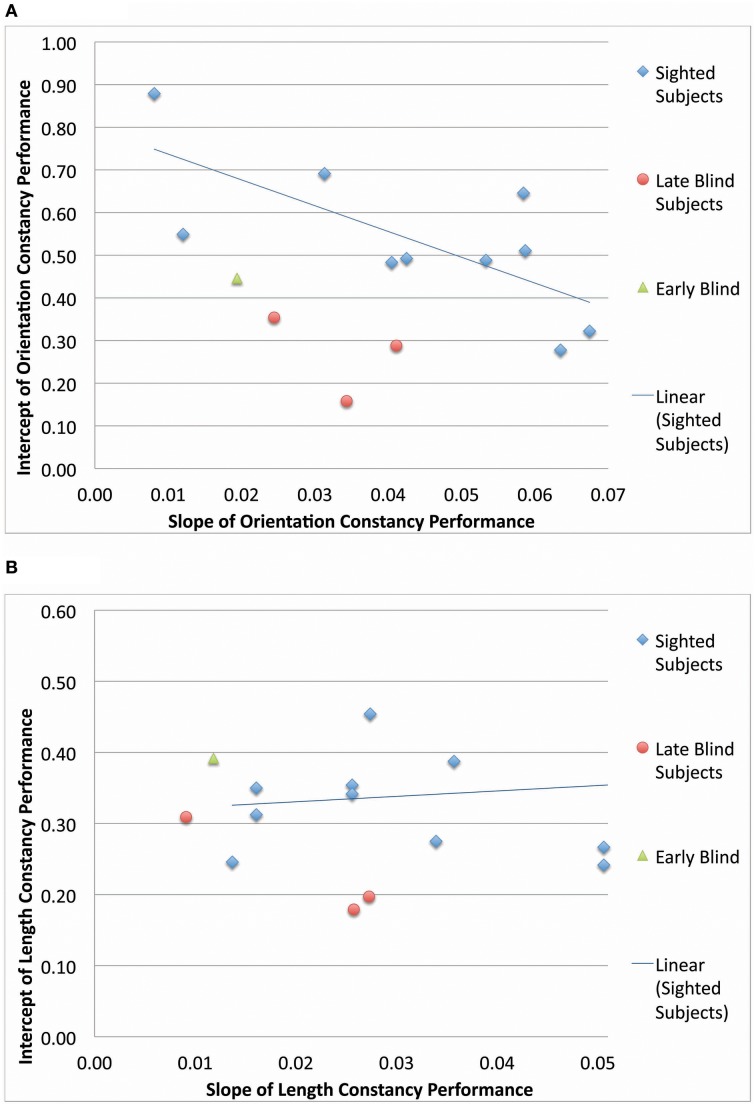
**A comparison between blind and sighted group performance**. The intercepts and slopes (from the performance as a function of time plots) for each subject are scatter plotted for orientation constancy **(A)** and for length constancy **(B)**.

Blind participants were divided into late and early blind categories, and their slopes of improvement and intercepts of learning curves were compared to those of the sighted participants (Figure 6). In both the length constancy and orientation constancy tasks, the early blind participant (*N* = 1) improved at the slowest rate (i.e., exhibited the smallest slope). The late blind participants (*N* = 3) improved at a faster rate, and the sighted (*N* = 9–10) improved at the fastest rate (i.e., exhibited the largest slope). However, there were no systematic differences across groups in terms of intercept. The sighted participants have the advantage of familiarity with visual principles (such as relative size), as well as visuomotor skills due to daily visual experience. These existing skills can be generalized when learning to process vOICe inputs visually or to relearn a constancy. The late blind have less recent visual experience than the sighted, as they have been visually deprived for years, if not decades. Finally, the early blind have no prior visual experience. Therefore, the rate of learning seems to correlate with visual experience; however, no definitive statement can be made at this point due to the low number of late blind (*N* = 3) and early blind participants (*N* = 1).

## Discussion

This study had the goal of not only training the sighted and blind on two 2D constancies but also understanding any training elements that aided in that learning. Overall, we found that the sighted and blind were able to identify line angles independent of head tilt and identify line length independent of line angle (2D orientation and length constancy, respectively). It was also determined that improvement at the length constancy task significantly correlated with head tilting movements in the sighted group. Time to perform the tasks decreased over training indicating a trend toward automaticity. These experiments not only have pointed toward the importance of sensory-motor integration but have also shown that constancy processing can be achieved with auditory SS.

The successful learning of constancies by early and late blind as well as sighted individuals highlights the value of the spatial perception of external objects. Constancies allow for the association of stimuli that have different proximal properties yet represent the same object or feature in real 3D space, thus making a cohesive representation of external objects. Constancy learning will likely improve performance with SS at daily tasks such as object avoidance or retrieval. Therefore, training on SS devices should incorporate forms of constancy learning, whether it is object recognition from multiple perspectives and lighting conditions, or object orientation estimation with head tilt and movement. These types of tasks are feasible to learn by means of SS (as shown in this study) and will enhance the capabilities of the blind using SS (as has been shown with the sighted in vision). Further, length and orientation constancy learning with SS demonstrate “externalization” and spatial perception (Rock, 1983), and are therefore critical first steps to breaking down complex natural scenes into objects and relative spatial positions with SS (a task that has proven to be difficult with SS).

The constancy learning in this paper adds to the current knowledge of SS perceptual learning. The SS field has explored recognition, localization, illusions, depth perception and more (Bach-Y-Rita et al., 1969; Renier et al., 2005; Auvray et al., 2007; Poirier et al., 2007a; Renier and De Volder, 2010; Brown and Proulx, 2013; Brown et al., 2014; Proulx et al., 2014), but mostly neglected the role of constancy learning in sensory perception, with the exception of anecdotal accounts (Bach-Y-Rita et al., 1969). This study begins to fill that void by studying two types of constancy: orientation and length, with sighted and blind participants (note that the number of blind participants is small in this study, which does constrain our conclusions). The SS field has also shown the value of hand-eye coordination and self-guided activities in learning perceptual principles. This study adds to those general principles by tying a specific movement type (head-tilt) with enhanced learning at particular constancy task (length constancy). Thus, we highlighted a step in the complex process of sensory integration, which supports and advances the general SS learning principles. It is by this type of dissection of the sensory-motor learning process that the SS field will learn how self-directed-movement can further improve SS learning.

Constancy learning with the vOICe device demonstrates a valuable learning ability and therefore likely plasticity of the adult brain. Several studies have indicated that visual cortical regions are recruited to process SS auditory signals. Tactile and auditory SS learning functionally recruits visual regions via extensive plasticity in blind and sighted users (Amedi et al., 2007; Poirier et al., 2007b; Merabet et al., 2009; Kupers et al., 2010). In particular, SS face stimuli activate the FFA (fusiform face area), SS shape discrimination activates the LOtv (lateral occipital tactile visual) area, and SS reading activates the VWFA (visual word form area) (Amedi et al., 2007; Plaza et al., 2009; Striem-Amit et al., 2012). In addition, repetitive TMS experiments have shown that congenital and late-blind users causally recruit visual regions for SS processing (Collignon et al., 2007; Merabet et al., 2009). Although no previous studies have been reported in which brain scans have been performed following constancy learning with SS, a broad network of regions from sub-cortical auditory areas, primary auditory regions, multimodal regions, and then visual regions may play a role. Furthermore, active sensory feedback among these regions and motor areas likely improves multisensory network efficacy. If multisensory experiences and feedback shape such a neural network, active, as opposed to passive or static, learning procedures may enhance network shaping.

It is revealing that the improved length constancy performance significantly correlated with head tilt (Figure 4). This is a critical and core finding of this study, as it indicates a more efficient SS training technique with additional sensorimotor interaction. It also indicates a key method for learning of constancy in vision as well as with SS.

**Figure 7:**
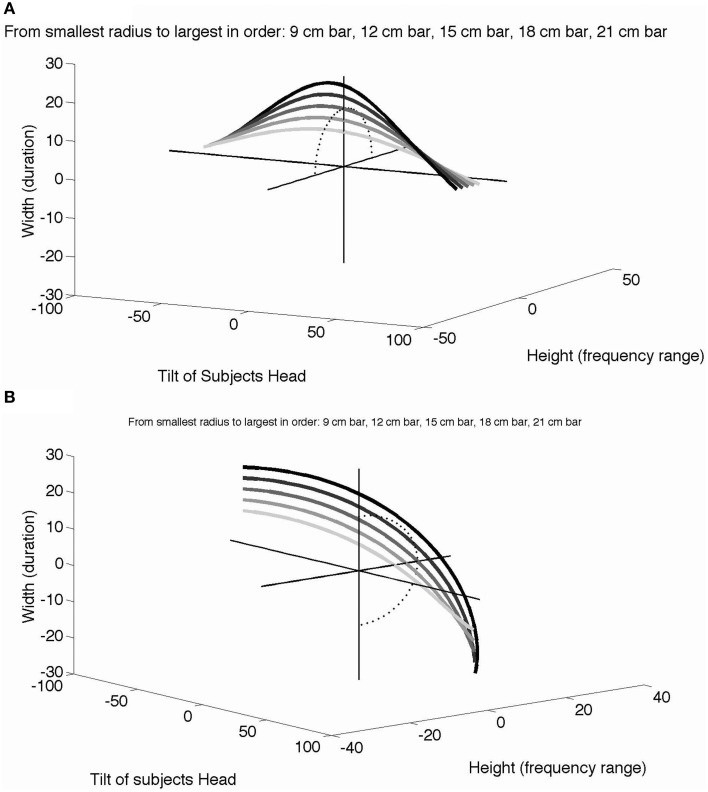
**The role of head tilt in length constancy**. A horizontal line stimulus **(A)** or vertical line stimulus **(B)** dynamically changes in horizontal width (duration of the sound) and vertical height (range of the sound pitch) as the participant tilts their head from vertical (no head tilt, tilt = 0°) to 90° left (negative tilt) or right (positive tilt). Each curve represents a different line length ranging from 9 to 21 cm in length. The sets of curves are viewed from different 3D perspectives in each case for clarity. A dotted half circle is drawn on the height-width plane to show the projection of the 15 cm curve (i.e., the shape of the 15 cm curve without head tilt or the x-axis) in both figures. Additional plot details in Video S1 and Video S2.

The benefit of head tilt in learning length constancy can be described in mathematical and psychophysical terms. As a participant spontaneously tilts their head, they alter the tilt of the camera attached to glasses on their head, thereby altering the perceived angle of the line. As a linear object of finite size is rotated, the projected height and width also change according to *Lsin*(θ) and *Lcos*(θ), in which *L* is the length of the line and θ is the head tilt relative to vertical. The projected vertical height (range of pitch in the SS device) and horizontal width (duration of sound) as a function of head tilt are plotted in Figure 7 for a line placed horizontally (Figure 7A) or vertically (Figure 7B). In this plot, the radius of each curve (section of a circle) represents the line length, which remains the same (i.e., is constant) across all the different head tilts and line angles. With multiple head tilts, the brain learns from the association of different points on each curve to identify a given linear object as one entity (i.e., a white bar of a particular length), and to interpret the different curves as each representing bars of different lengths. Learning general length constancy (not just the bars used in these experiments but all bar lengths oriented at different angles) requires similar exploration with head tilt, as well as similar associations of different input patterns that can be identified as the same real-world object. Furthermore, participants can begin to associate all of the stimulus cases (i.e., 0, 90, 45, and −45°) for a given line length, which in effect provide orientation-invariance and correspond to the object identity (line length). Obviously, active head-tilting and its sensory feedback have a critical role in such a dynamic associative learning of length constancy, specifically in SS but more generally in vision.

By tilting their head, the observer receives dynamic yet systematic changes of input parameters, as illustrated in Figures 7A,B. In effect, learning aims to identify all the data points within each curve as an “identical horizontal line,” at the same time discriminating among different curves as “lines of different lengths.” Intuitively, it would be much easier if the brain were able to compare a given entire curve to another in the graph using head tilt to move along the curve, as opposed to a point-by-point comparison in a set of (static) parameters. One may easily implement this more computationally in terms of S/N ratios in a Bayesian or MLE framework.

Modern neural computation models indicate that the brain is associative (Dayan and Abbott, 2001), using synaptic weighting to correlate related properties among neurons that “recognize” objects (Quiroga et al., 2005). The association of lines that are oriented at different angles but have constant length could be based on this type of neural processing. The enhanced learning due to head tilt (and retained in no-head-tilt trials) is consistent with neural network behavior using supervised learning. In supervised learning, a neural network improves at classification as more training images and correct answer pairs are presented (Dayan and Abbott, 2001). In length constancy training, with no-head-tilt in each trial, the number of trials is equal to the number of unique training images presented to the neural network. But when the participant uses head tilt, the number of unique training stimuli presented increases by a significant factor because each head position provides a unique training stimulus. Each head position presents different parameters to calculate length, thereby effectively increasing the number of training stimuli presented during supervised learning greatly and making classification more accurate (Changizi et al., 2008).

The use of sensorimotor integration has been shown in this study to be important to learning with the vOICe device (Epstein et al., 1989; Segond et al., 2005; Poirier et al., 2006; Proulx et al., 2008; Siegle and Warren, 2010; Levy-Tzedek et al., 2012; Haigh et al., 2013). It is important for further device design and use to discuss how broadly this finding applies to different SS devices with tactile or auditory interfaces. Despite variations in visual-to-auditory or tactile encodings most SS devices have a head mounted camera for dynamically “viewing” the environment. The head-tilt based learning improvement would likely work for all of the SS devices with a head-mounted camera. While the vOICe has a dramatically different encoding of line length vertically (frequency range) *vs*. horizontally (sound duration), this is not a critical feature to improvement with head-tilt. Given our analyses, it is most likely that head-tilt is not a method to view the line at the easiest angle to interpret it with vOICe (especially since improvement was maintained even when head-tilt was prohibited in the final training sessions), but rather a sensory-motor engagement that would be useful even if the encoding is same in all directions (such as with the tactile devices). Therefore, our result of sensorimotor learning with length constancy is likely moderately generalizable to other SS devices.

In summary, critical perceptual properties such as constancy and externalization can be achieved to some degree with current SS devices. This implies that vision-like processing, or perception beyond sensation, can be attained with SS. Furthermore, dynamic interaction with stimuli is shown to be critical to learning with sensory substitution due to sensory-motor engagement.

## Author contributions

The study design and concept were generated by NS and SS. Subject training and testing was performed by NS and YZ. NS and SS drafted the manuscript; YZ reviewed and commented on the manuscript.

### Conflict of interest statement

The authors declare that the research was conducted in the absence of any commercial or financial relationships that could be construed as a potential conflict of interest.

## Supplementary material

Video S1 and S2 **This is a short video demonstration of the dynamic change in vOICe encoding of a horizontal bar (A) or vertical bar (B) when viewed with head tilting during the length constancy task**.
